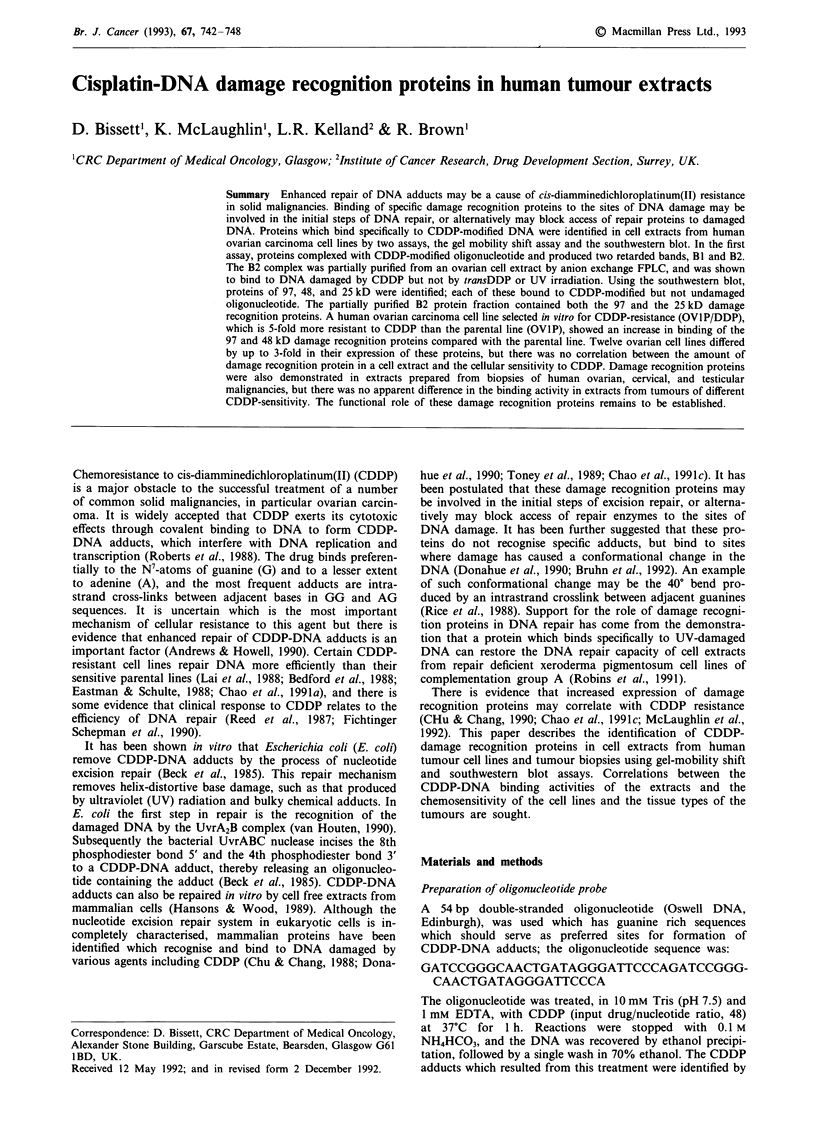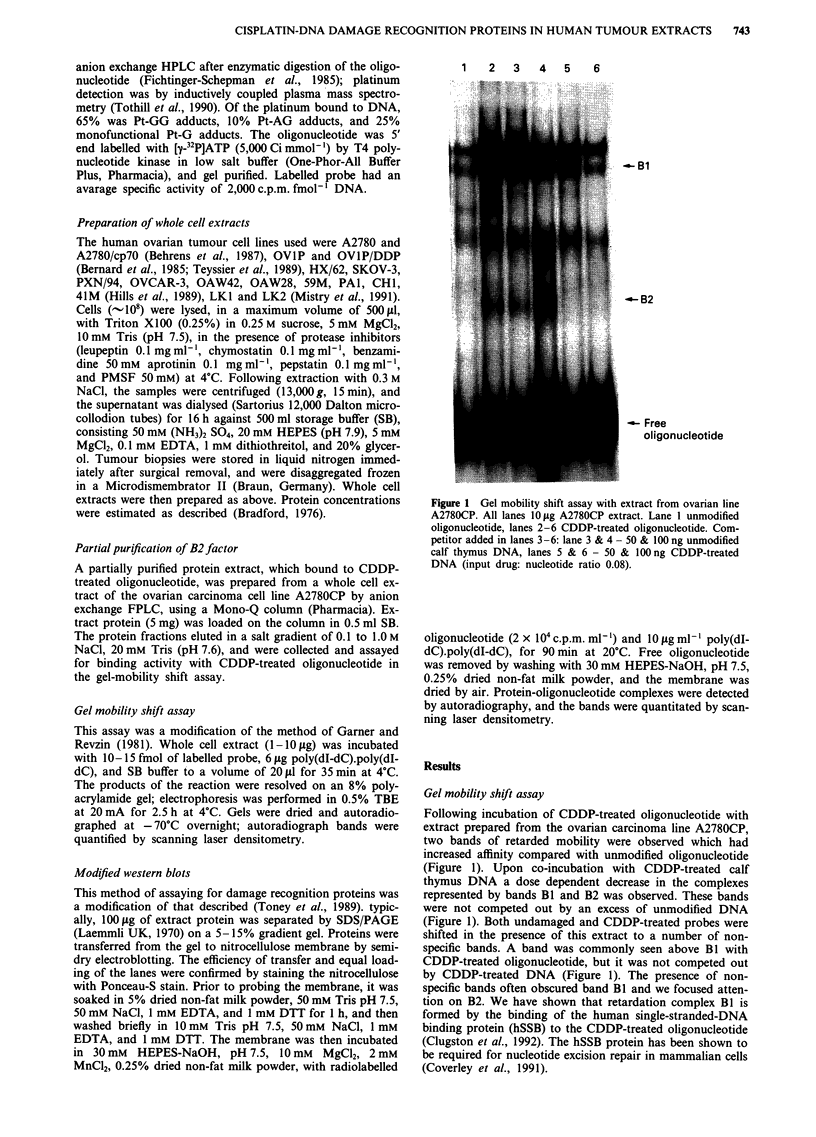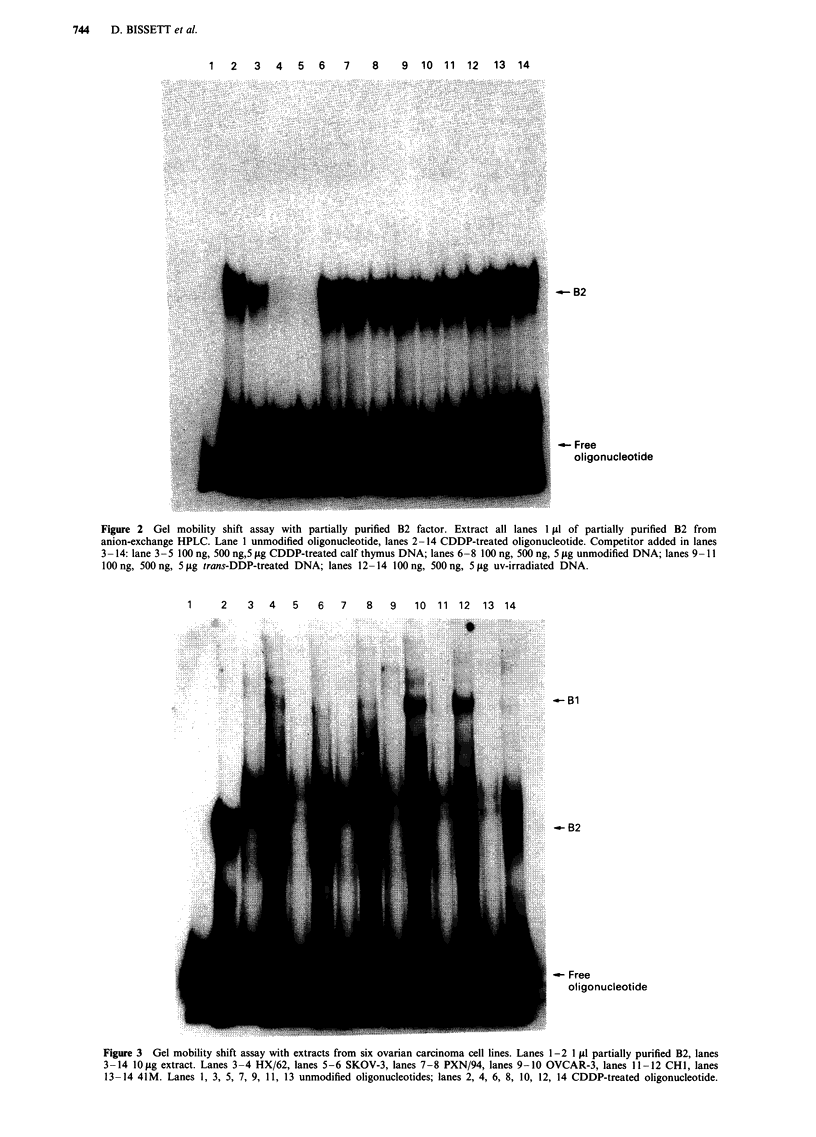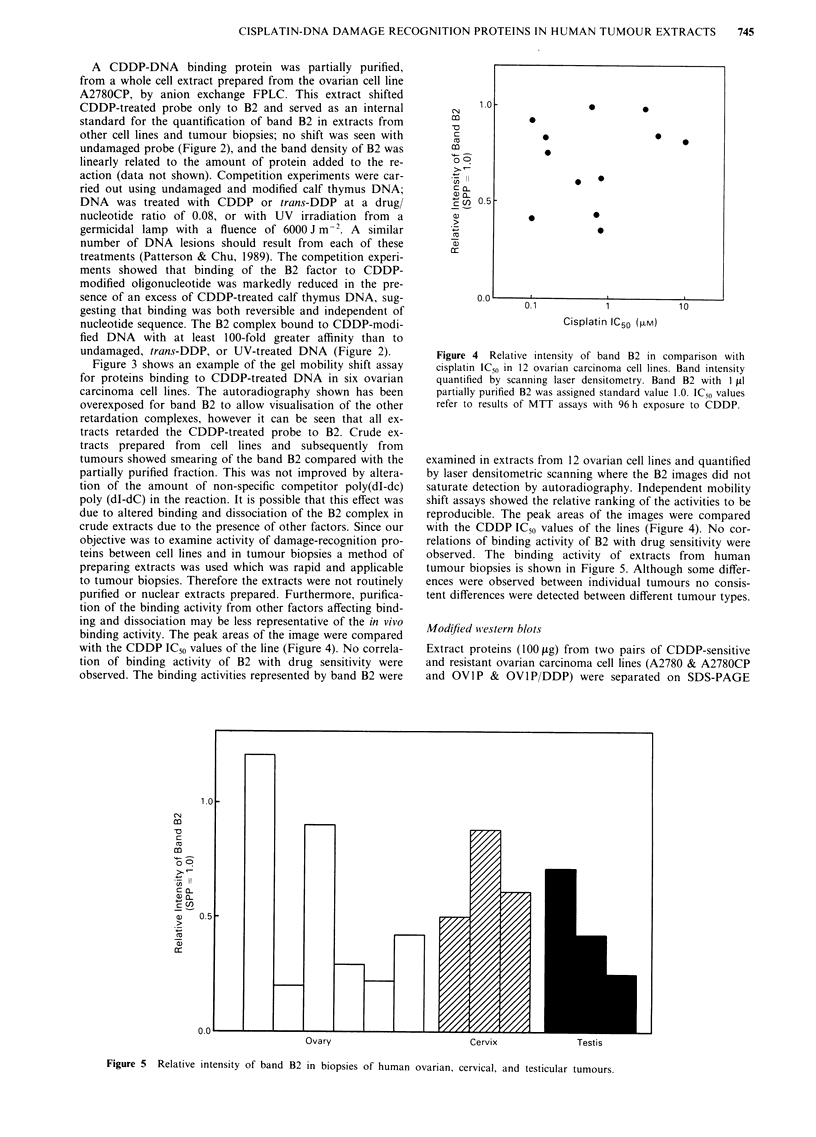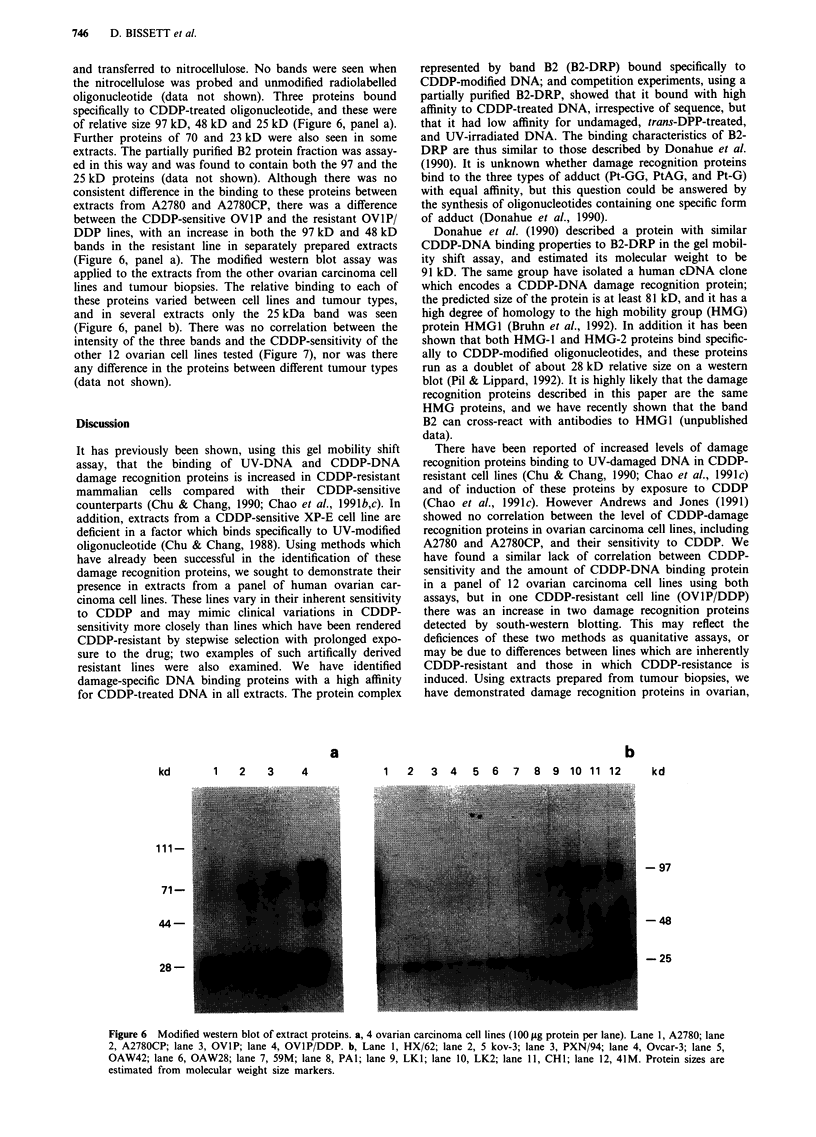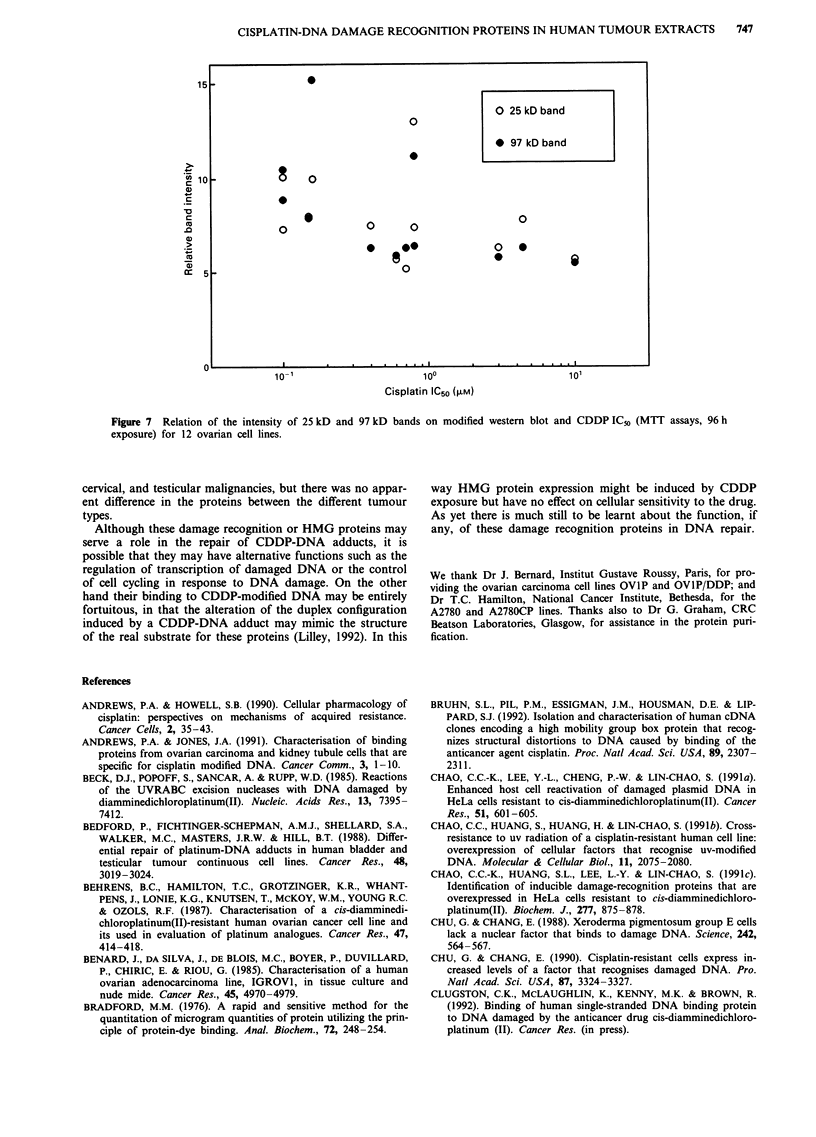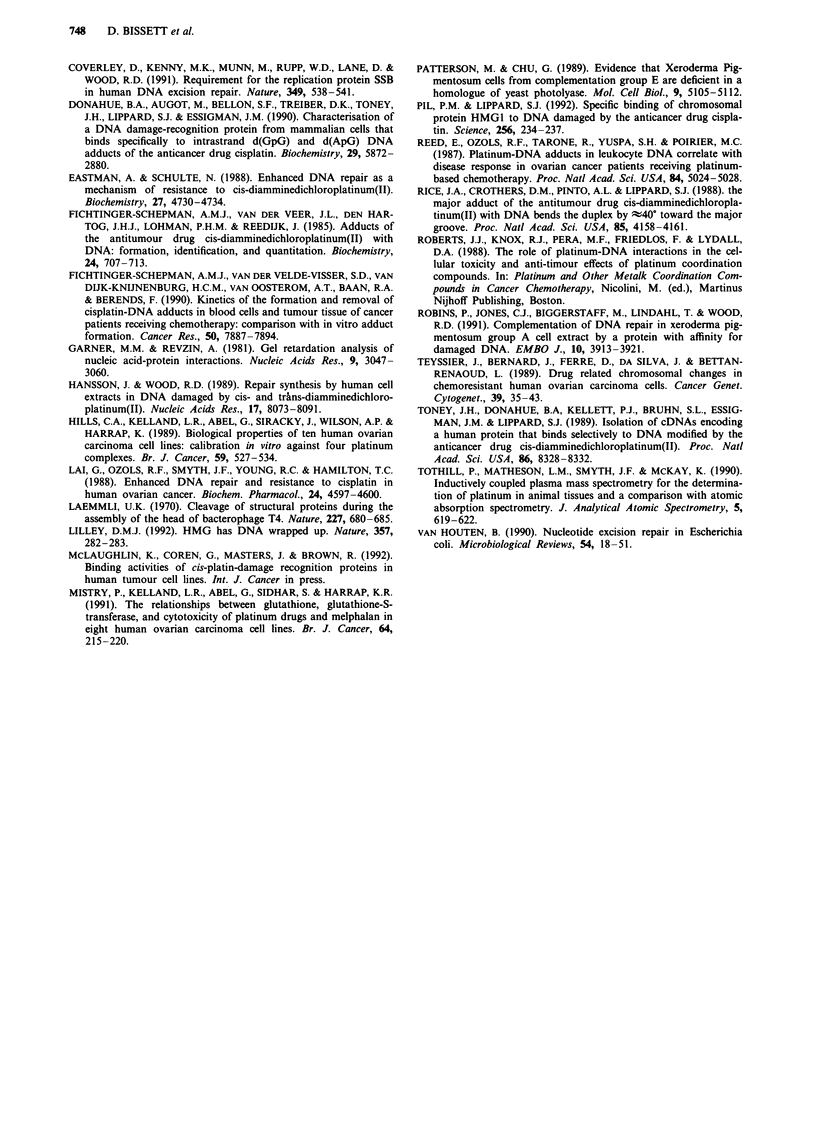# Cisplatin-DNA damage recognition proteins in human tumour extracts.

**DOI:** 10.1038/bjc.1993.135

**Published:** 1993-04

**Authors:** D. Bissett, K. McLaughlin, L. R. Kelland, R. Brown

**Affiliations:** CRC Department of Medical Oncology, Glasgow, UK.

## Abstract

**Images:**


Br. J. Cancer (1993), 67, 742–748 © Macmillan Press Ltd., 1993

### Cisplatin-DNA damage recognition proteins in human tumour extracts

D. Bissett[1], K. McLaughlin[1], L.R. Kelland[2] & R. Brown[1]

[1]*CRC Department of Medical Oncology, Glasgow;* [2]*Institute of Cancer Research, Drug Development Section, Surrey, UK.*

**Summary** Enhanced repair of DNA adducts may be a cause of *cis*-diamminedichloroplatinum(II) resistance in solid malignancies. Binding of specific damage recognition proteins to the sites of DNA damage may be involved in the initial steps of DNA repair, or alternatively may block access of repair proteins to damaged DNA. Proteins which bind specifically to CDDP-modified DNA were identified in cell extracts from human ovarian carcinoma cell lines by two assays, the gel mobility shift assay and the southwestern blot. In the first assay, proteins complexed with CDDP-modified oligonucleotide and produced two retarded bands, B1 and B2. The B2 complex was partially purified from an ovarian cell extract by anion exchange FPLC, and was shown to bind to DNA damaged by CDDP but not by *trans*DDP or UV irradiation. Using the southwestern blot, proteins of 97, 48, and 25 kD were identified; each of these bound to CDDP-modified but not undamaged oligonucleotide. The partially purified B2 protein fraction contained both the 97 and the 25 kD damage recognition proteins. A human ovarian carcinoma cell line selected *in vitro* for CDDP-resistance (OV1P/DDP), which is 5-fold more resistant to CDDP than the parental line (OV1P), showed an increase in binding of the 97 and 48 kD damage recognition proteins compared with the parental line. Twelve ovarian cell lines differed by up to 3-fold in their expression of these proteins, but there was no correlation between the amount of damage recognition protein in a cell extract and the cellular sensitivity to CDDP. Damage recognition proteins were also demonstrated in extracts prepared from biopsies of human ovarian, cervical, and testicular malignancies, but there was no apparent difference in the binding activity in extracts from tumours of different CDDP-sensitivity. The functional role of these damage recognition proteins remains to be established.

Chemoresistance to cis-diamminedichloroplatinum(II) (CDDP) is a major obstacle to the successful treatment of a number of common solid malignancies, in particular ovarian carcinoma. It is widely accepted that CDDP exerts its cytotoxic effects through covalent binding to DNA to form CDDP-DNA adducts, which interfere with DNA replication and transcription (Roberts *et al.*, 1988). The drug binds preferentially to the $N^7$-atoms of guanine (G) and to a lesser extent to adenine (A), and the most frequent adducts are intrastrand cross-links between adjacent bases in GG and AG sequences. It is uncertain which is the most important mechanism of cellular resistance to this agent but there is evidence that enhanced repair of CDDP-DNA adducts is an important factor (Andrews & Howell, 1990). Certain CDDP-resistant cell lines repair DNA more efficiently than their sensitive parental lines (Lai *et al.*, 1988; Bedford *et al.*, 1988; Eastman & Schulte, 1988; Chao *et al.*, 1991*a*), and there is some evidence that clinical response to CDDP relates to the efficiency of DNA repair (Reed *et al.*, 1987; Fichtinger Schepman *et al.*, 1990).

It has been shown *in vitro* that *Escherichia coli* (*E. coli*) remove CDDP-DNA adducts by the process of nucleotide excision repair (Beck *et al.*, 1985). This repair mechanism removes helix-distortive base damage, such as that produced by ultraviolet (UV) radiation and bulky chemical adducts. In *E. coli* the first step in repair is the recognition of the damaged DNA by the $UvrA_2B$ complex (van Houten, 1990). Subsequently the bacterial UvrABC nuclease incises the 8th phosphodiester bond 5′ and the 4th phosphodiester bond 3′ to a CDDP-DNA adduct, thereby releasing an oligonucleotide containing the adduct (Beck *et al.*, 1985). CDDP-DNA adducts can also be repaired *in vitro* by cell free extracts from mammalian cells (Hansons & Wood, 1989). Although the nucleotide excision repair system in eukaryotic cells is incompletely characterised, mammalian proteins have been identified which recognise and bind to DNA damaged by various agents including CDDP (Chu & Chang, 1988; Donahue *et al.*, 1990; Toney *et al.*, 1989; Chao *et al.*, 1991*c*). It has been postulated that these damage recognition proteins may be involved in the initial steps of excision repair, or alternatively may block access of repair enzymes to the sites of DNA damage. It has been further suggested that these proteins do not recognise specific adducts, but bind to sites where damage has caused a conformational change in the DNA (Donahue *et al.*, 1990; Bruhn *et al.*, 1992). An example of such conformational change may be the 40° bend produced by an intrastrand crosslink between adjacent guanines (Rice *et al.*, 1988). Support for the role of damage recognition proteins in DNA repair has come from the demonstration that a protein which binds specifically to UV-damaged DNA can restore the DNA repair capacity of cell extracts from repair deficient xeroderma pigmentosum cell lines of complementation group A (Robins *et al.*, 1991).

There is evidence that increased expression of damage recognition proteins may correlate with CDDP resistance (CHu & Chang, 1990; Chao *et al.*, 1991*c*; McLaughlin *et al.*, 1992). This paper describes the identification of CDDP-damage recognition proteins in cell extracts from human tumour cell lines and tumour biopsies using gel-mobility shift and southwestern blot assays. Correlations between the CDDP-DNA binding activities of the extracts and the chemosensitivity of the cell lines and the tissue types of the tumours are sought.

### Materials and methods

### *Preparation of oligonucleotide probe*

A 54 bp double-stranded oligonucleotide (Oswell DNA, Edinburgh), was used which has guanine rich sequences which should serve as preferred sites for formation of CDDP-DNA adducts; the oligonucleotide sequence was:

GATCCGGGCAACTGATAGGGATTCCCAGATCCGGG-CAACTGATAGGGATTCCCA

The oligonucleotide was treated, in 10 mM Tris (pH 7.5) and 1 mM EDTA, with CDDP (input drug/nucleotide ratio, 48) at 37°C for 1 h. Reactions were stopped with 0.1 M $NH_4HCO_3$, and the DNA was recovered by ethanol precipitation, followed by a single wash in 70% ethanol. The CDDP adducts which resulted from this treatment were identified by

Correspondence: D. Bissett, CRC Department of Medical Oncology, Alexander Stone Building, Garscube Estate, Bearsden, Glasgow G61 1BD, UK.
Received 12 May 1992; and in revised form 2 December 1992.

anion exchange HPLC after enzymatic digestion of the oligonucleotide (Fichtinger-Schepman *et al.*, 1985); platinum detection was by inductively coupled plasma mass spectrometry (Tothill *et al.*, 1990). Of the platinum bound to DNA, 65% was Pt-GG adducts, 10% Pt-AG adducts, and 25% monofunctional Pt-G adducts. The oligonucleotide was 5′ end labelled with [$\gamma$-$^{32}$P]ATP (5,000 Ci mmol$^{-1}$) by T4 polynucleotide kinase in low salt buffer (One-Phor-All Buffer Plus, Pharmacia), and gel purified. Labelled probe had an avarage specific activity of 2,000 c.p.m. fmol$^{-1}$ DNA.

*Preparation of whole cell extracts*

The human ovarian tumour cell lines used were A2780 and A2780/cp70 (Behrens *et al.*, 1987), OV1P and OV1P/DDP (Bernard *et al.*, 1985; Teyssier *et al.*, 1989), HX/62, SKOV-3, PXN/94, OVCAR-3, OAW42, OAW28, 59M, PA1, CH1, 41M (Hills *et al.*, 1989), LK1 and LK2 (Mistry *et al.*, 1991). Cells ($\sim10^8$) were lysed, in a maximum volume of 500 µl, with Triton X100 (0.25%) in 0.25 M sucrose, 5 mM $MgCl_2$, 10 mM Tris (pH 7.5), in the presence of protease inhibitors (leupeptin 0.1 mg ml$^{-1}$, chymostatin 0.1 mg ml$^{-1}$, benzamidine 50 mM aprotinin 0.1 mg ml$^{-1}$, pepstatin 0.1 mg ml$^{-1}$, and PMSF 50 mM) at 4°C. Following extraction with 0.3 M NaCl, the samples were centrifuged (13,000 *g*, 15 min), and the supernatant was dialysed (Sartorius 12,000 Dalton microcollodion tubes) for 16 h against 500 ml storage buffer (SB), consisting 50 mM $(NH_3)_2$ $SO_4$, 20 mM HEPES (pH 7.9), 5 mM $MgCl_2$, 0.1 mM EDTA, 1 mM dithiothreitol, and 20% glycerol. Tumour biopsies were stored in liquid nitrogen immediately after surgical removal, and were disaggregated frozen in a Microdismembrator II (Braun, Germany). Whole cell extracts were then prepared as above. Protein concentrations were estimated as described (Bradford, 1976).

*Partial purification of B2 factor*

A partially purified protein extract, which bound to CDDP-treated oligonucleotide, was prepared from a whole cell extract of the ovarian carcinoma cell line A2780CP by anion exchange FPLC, using a Mono-Q column (Pharmacia). Extract protein (5 mg) was loaded on the column in 0.5 ml SB. The protein fractions eluted in a salt gradient of 0.1 to 1.0 M NaCl, 20 mM Tris (pH 7.6), and were collected and assayed for binding activity with CDDP-treated oligonucleotide in the gel-mobility shift assay.

*Gel mobility shift assay*

This assay was a modification of the method of Garner and Revzin (1981). Whole cell extract (1–10 µg) was incubated with 10–15 fmol of labelled probe, 6 µg poly(dI-dC).poly(dI-dC), and SB buffer to a volume of 20 µl for 35 min at 4°C. The products of the reaction were resolved on an 8% polyacrylamide gel; electrophoresis was performed in 0.5% TBE at 20 mA for 2.5 h at 4°C. Gels were dried and autoradiographed at −70°C overnight; autoradiograph bands were quantified by scanning laser densitometry.

*Modified western blots*

This method of assaying for damage recognition proteins was a modification of that described (Toney *et al.*, 1989). typically, 100 µg of extract protein was separated by SDS/PAGE (Laemmli UK, 1970) on a 5–15% gradient gel. Proteins were transferred from the gel to nitrocellulose membrane by semidry electroblotting. The efficiency of transfer and equal loading of the lanes were confirmed by staining the nitrocellulose with Ponceau-S stain. Prior to probing the membrane, it was soaked in 5% dried non-fat milk powder, 50 mM Tris pH 7.5, 50 mM NaCl, 1 mM EDTA, and 1 mM DTT for 1 h, and then washed briefly in 10 mM Tris pH 7.5, 50 mM NaCl, 1 mM EDTA, and 1 mM DTT. The membrane was then incubated in 30 mM HEPES-NaOH, pH 7.5, 10 mM $MgCl_2$, 2 mM $MnCl_2$, 0.25% dried non-fat milk powder, with radiolabelled

**Figure 1** Gel mobility shift assay with extract from ovarian line A2780CP. All lanes 10 µg A2780CP extract. Lane 1 unmodified oligonucleotide, lanes 2–6 CDDP-treated oligonucleotide. Competitor added in lanes 3–6: lane 3 & 4 – 50 & 100 ng unmodified calf thymus DNA, lanes 5 & 6 – 50 & 100 ng CDDP-treated DNA (input drug: nucleotide ratio 0.08).

oligonucleotide ($2\times10^4$ c.p.m. ml$^{-1}$) and 10 µg ml$^{-1}$ poly(dI-dC).poly(dI-dC), for 90 min at 20°C. Free oligonucleotide was removed by washing with 30 mM HEPES-NaOH, pH 7.5, 0.25% dried non-fat milk powder, and the membrane was dried by air. Protein-oligonucleotide complexes were detected by autoradiography, and the bands were quantitated by scanning laser densitometry.

### Results

*Gel mobility shift assay*

Following incubation of CDDP-treated oligonucleotide with extract prepared from the ovarian carcinoma line A2780CP, two bands of retarded mobility were observed which had increased affinity compared with unmodified oligonucleotide (Figure 1). Upon co-incubation with CDDP-treated calf thymus DNA a dose dependent decrease in the complexes represented by bands B1 and B2 was observed. These bands were not competed out by an excess of unmodified DNA (Figure 1). Both undamaged and CDDP-treated probes were shifted in the presence of this extract to a number of non-specific bands. A band was commonly seen above B1 with CDDP-treated oligonucleotide, but it was not competed out by CDDP-treated DNA (Figure 1). The presence of non-specific bands often obscured band B1 and we focused attention on B2. We have shown that retardation complex B1 is formed by the binding of the human single-stranded-DNA binding protein (hSSB) to the CDDP-treated oligonucleotide (Clugston *et al.*, 1992). The hSSB protein has been shown to be required for nucleotide excision repair in mammalian cells (Coverley *et al.*, 1991).

**Figure 2** Gel mobility shift assay with partially purified B2 factor. Extract all lanes 1 µl of partially purified B2 from anion-exchange HPLC. Lane 1 unmodified oligonucleotide, lanes 2–14 CDDP-treated oligonucleotide. Competitor added in lanes 3–14: lane 3–5 100 ng, 500 ng,5 µg CDDP-treated calf thymus DNA; lanes 6–8 100 ng, 500 ng, 5 µg unmodified DNA; lanes 9–11 100 ng, 500 ng, 5 µg *trans*-DDP-treated DNA; lanes 12–14 100 ng, 500 ng, 5 µg uv-irradiated DNA.

**Figure 3** Gel mobility shift assay with extracts from six ovarian carcinoma cell lines. Lanes 1–2 1 µl partially purified B2, lanes 3–14 10 µg extract. Lanes 3–4 HX/62, lanes 5–6 SKOV-3, lanes 7–8 PXN/94, lanes 9–10 OVCAR-3, lanes 11–12 CH1, lanes 13–14 41M. Lanes 1, 3, 5, 7, 9, 11, 13 unmodified oligonucleotides; lanes 2, 4, 6, 8, 10, 12, 14 CDDP-treated oligonucleotide.

A CDDP-DNA binding protein was partially purified, from a whole cell extract prepared from the ovarian cell line A2780CP, by anion exchange FPLC. This extract shifted CDDP-treated probe only to B2 and served as an internal standard for the quantification of band B2 in extracts from other cell lines and tumour biopsies; no shift was seen with undamaged probe (Figure 2), and the band density of B2 was linearly related to the amount of protein added to the reaction (data not shown). Competition experiments were carried out using undamaged and modified calf thymus DNA; DNA was treated with CDDP or *trans*-DDP at a drug/nucleotide ratio of 0.08, or with UV irradiation from a germicidal lamp with a fluence of 6000 J $m^{-2}$. A similar number of DNA lesions should result from each of these treatments (Patterson & Chu, 1989). The competition experiments showed that binding of the B2 factor to CDDP-modified oligonucleotide was markedly reduced in the presence of an excess of CDDP-treated calf thymus DNA, suggesting that binding was both reversible and independent of nucleotide sequence. The B2 complex bound to CDDP-modified DNA with at least 100-fold greater affinity than to undamaged, *trans*-DDP, or UV-treated DNA (Figure 2).

Figure 3 shows an example of the gel mobility shift assay for proteins binding to CDDP-treated DNA in six ovarian carcinoma cell lines. The autoradiography shown has been overexposed for band B2 to allow visualisation of the other retardation complexes, however it can be seen that all extracts retarded the CDDP-treated probe to B2. Crude extracts prepared from cell lines and subsequently from tumours showed smearing of the band B2 compared with the partially purified fraction. This was not improved by alteration of the amount of non-specific competitor poly(dI-dc) poly (dI-dC) in the reaction. It is possible that this effect was due to altered binding and dissociation of the B2 complex in crude extracts due to the presence of other factors. Since our objective was to examine activity of damage-recognition proteins between cell lines and in tumour biopsies a method of preparing extracts was used which was rapid and applicable to tumour biopsies. Therefore the extracts were not routinely purified or nuclear extracts prepared. Furthermore, purification of the binding activity from other factors affecting binding and dissociation may be less representative of the *in vivo* binding activity. The peak areas of the image were compared with the CDDP $IC_{50}$ values of the line (Figure 4). No correlation of binding activity of B2 with drug sensitivity were observed. The binding activities represented by band B2 were examined in extracts from 12 ovarian cell lines and quantified by laser densitometric scanning where the B2 images did not saturate detection by autoradiography. Independent mobility shift assays showed the relative ranking of the activities to be reproducible. The peak areas of the images were compared with the CDDP $IC_{50}$ values of the lines (Figure 4). No correlations of binding activity of B2 with drug sensitivity were observed. The binding activity of extracts from human tumour biopsies is shown in Figure 5. Although some differences were observed between individual tumours no consistent differences were detected between different tumour types.

**Figure 4** Relative intensity of band B2 in comparison with cisplatin $IC_{50}$ in 12 ovarian carcinoma cell lines. Band intensity quantified by scanning laser densitometry. Band B2 with 1 μl partially purified B2 was assigned standard value 1.0. $IC_{50}$ values refer to results of MTT assays with 96 h exposure to CDDP.

### *Modified western blots*

Extract proteins (100 μg) from two pairs of CDDP-sensitive and resistant ovarian carcinoma cell lines (A2780 & A2780CP and OV1P & OV1P/DDP) were separated on SDS-PAGE

**Figure 5** Relative intensity of band B2 in biopsies of human ovarian, cervical, and testicular tumours.

and transferred to nitrocellulose. No bands were seen when the nitrocellulose was probed and unmodified radiolabelled oligonucleotide (data not shown). Three proteins bound specifically to CDDP-treated oligonucleotide, and these were of relative size 97 kD, 48 kD and 25 kD (Figure 6, panel a). Further proteins of 70 and 23 kD were also seen in some extracts. The partially purified B2 protein fraction was assayed in this way and was found to contain both the 97 and the 25 kD proteins (data not shown). Although there was no consistent difference in the binding to these proteins between extracts from A2780 and A2780CP, there was a difference between the CDDP-sensitive OV1P and the resistant OV1P/DDP lines, with an increase in both the 97 kD and 48 kD bands in the resistant line in separately prepared extracts (Figure 6, panel a). The modified western blot assay was applied to the extracts from the other ovarian carcinoma cell lines and tumour biopsies. The relative binding to each of these proteins varied between cell lines and tumour types, and in several extracts only the 25 kDa band was seen (Figure 6, panel b). There was no correlation between the intensity of the three bands and the CDDP-sensitivity of the other 12 ovarian cell lines tested (Figure 7), nor was there any difference in the proteins between different tumour types (data not shown).

### Discussion

It has previously been shown, using this gel mobility shift assay, that the binding of UV-DNA and CDDP-DNA damage recognition proteins is increased in CDDP-resistant mammalian cells compared with their CDDP-sensitive counterparts (Chu & Chang, 1990; Chao *et al.*, 1991*b*,*c*). In addition, extracts from a CDDP-sensitive XP-E cell line are deficient in a factor which binds specifically to UV-modified oligonucleotide (Chu & Chang, 1988). Using methods which have already been successful in the identification of these damage recognition proteins, we sought to demonstrate their presence in extracts from a panel of human ovarian carcinoma cell lines. These lines vary in their inherent sensitivity to CDDP and may mimic clinical variations in CDDP-sensitivity more closely than lines which have been rendered CDDP-resistant by stepwise selection with prolonged exposure to the drug; two examples of such artifically derived resistant lines were also examined. We have identified damage-specific DNA binding proteins with a high affinity for CDDP-treated DNA in all extracts. The protein complex represented by band B2 (B2-DRP) bound specifically to CDDP-modified DNA; and competition experiments, using a partially purified B2-DRP, showed that it bound with high affinity to CDDP-treated DNA, irrespective of sequence, but that it had low affinity for undamaged, *trans*-DPP-treated, and UV-irradiated DNA. The binding characteristics of B2-DRP are thus similar to those described by Donahue *et al.* (1990). It is unknown whether damage recognition proteins bind to the three types of adduct (Pt-GG, PtAG, and Pt-G) with equal affinity, but this question could be answered by the synthesis of oligonucleotides containing one specific form of adduct (Donahue *et al.*, 1990).

Donahue *et al.* (1990) described a protein with similar CDDP-DNA binding properties to B2-DRP in the gel mobility shift assay, and estimated its molecular weight to be 91 kD. The same group have isolated a human cDNA clone which encodes a CDDP-DNA damage recognition protein; the predicted size of the protein is at least 81 kD, and it has a high degree of homology to the high mobility group (HMG) protein HMG1 (Bruhn *et al.*, 1992). In addition it has been shown that both HMG-1 and HMG-2 proteins bind specifically to CDDP-modified oligonucleotides, and these proteins run as a doublet of about 28 kD relative size on a western blot (Pil & Lippard, 1992). It is highly likely that the damage recognition proteins described in this paper are the same HMG proteins, and we have recently shown that the band B2 can cross-react with antibodies to HMG1 (unpublished data).

There have been reported of increased levels of damage recognition proteins binding to UV-damaged DNA in CDDP-resistant cell lines (Chu & Chang, 1990; Chao *et al.*, 1991*c*) and of induction of these proteins by exposure to CDDP (Chao *et al.*, 1991*c*). However Andrews and Jones (1991) showed no correlation between the level of CDDP-damage recognition proteins in ovarian carcinoma cell lines, including A2780 and A2780CP, and their sensitivity to CDDP. We have found a similar lack of correlation between CDDP-sensitivity and the amount of CDDP-DNA binding protein in a panel of 12 ovarian carcinoma cell lines using both assays, but in one CDDP-resistant cell line (OV1P/DDP) there was an increase in two damage recognition proteins detected by south-western blotting. This may reflect the deficiences of these two methods as quanitative assays, or may be due to differences between lines which are inherently CDDP-resistant and those in which CDDP-resistance is induced. Using extracts prepared from tumour biopsies, we have demonstrated damage recognition proteins in ovarian,

**Figure 6** Modified western blot of extract proteins. **a**, 4 ovarian carcinoma cell lines (100 μg protein per lane). Lane 1, A2780; lane 2, A2780CP; lane 3, OV1P; lane 4, OV1P/DDP. **b**, Lane 1, HX/62; lane 2, 5 kov-3; lane 3, PXN/94; lane 4, Ovcar-3; lane 5, OAW42; lane 6, OAW28; lane 7, 59M; lane 8, PA1; lane 9, LK1; lane 10, LK2; lane 11, CH1; lane 12, 41M. Protein sizes are estimated from molecular weight size markers.

**Figure 7** Relation of the intensity of 25 kD and 97 kD bands on modified western blot and CDDP $IC_{50}$ (MTT assays, 96 h exposure) for 12 ovarian cell lines.

cervical, and testicular malignancies, but there was no apparent difference in the proteins between the different tumour types.

Although these damage recognition or HMG proteins may serve a role in the repair of CDDP-DNA adducts, it is possible that they may have alternative functions such as the regulation of transcription of damaged DNA or the control of cell cycling in response to DNA damage. On the other hand their binding to CDDP-modified DNA may be entirely fortuitous, in that the alteration of the duplex configuration induced by a CDDP-DNA adduct may mimic the structure of the real substrate for these proteins (Lilley, 1992). In this way HMG protein expression might be induced by CDDP exposure but have no effect on cellular sensitivity to the drug. As yet there is much still to be learnt about the function, if any, of these damage recognition proteins in DNA repair.

We thank Dr J. Bernard, Institut Gustave Roussy, Paris, for providing the ovarian carcinoma cell lines OV1P and OV1P/DDP; and Dr T.C. Hamilton, National Cancer Institute, Bethesda, for the A2780 and A2780CP lines. Thanks also to Dr G. Graham, CRC Beatson Laboratories, Glasgow, for assistance in the protein purification.

### References

ANDREWS, P.A. & HOWELL, S.B. (1990). Cellular pharmacology of cisplatin: perspectives on mechanisms of acquired resistance. *Cancer Cells*, **2**, 35–43.

ANDREWS, P.A. & JONES, J.A. (1991). Characterisation of binding proteins from ovarian carcinoma and kidney tubule cells that are specific for cisplatin modified DNA. *Cancer Comm.*, **3**, 1–10.

BECK, D.J., POPOFF, S., SANCAR, A. & RUPP, W.D. (1985). Reactions of the UVRABC excision nucleases with DNA damaged by diamminedichloroplatinum(II). *Nucleic. Acids Res.*, **13**, 7395–7412.

BEDFORD, P., FICHTINGER-SCHEPMAN, A.M.J., SHELLARD, S.A., WALKER, M.C., MASTERS, J.R.W. & HILL, B.T. (1988). Differential repair of platinum-DNA adducts in human bladder and testicular tumour continuous cell lines. *Cancer Res.*, **48**, 3019–3024.

BEHRENS, B.C., HAMILTON, T.C., GROTZINGER, K.R., WHANT-PENS, J., LONIE, K.G., KNUTSEN, T., MCKOY, W.M., YOUNG R.C. & OZOLS, R.F. (1987). Characterisation of a *cis*-diamminedichloroplatinum(II)-resistant human ovarian cancer cell line and its used in evaluation of platinum analogues. *Cancer Res.*, **47**, 414–418.

BENARD, J., DA SILVA, J., DE BLOIS, M.C., BOYER, P., DUVILLARD, P., CHIRIC, E. & RIOU, G. (1985). Characterisation of a human ovarian adenocarcinoma line, IGROV1, in tissue culture and nude mide. *Cancer Res.*, **45**, 4970–4979.

BRADFORD, M.M. (1976). A rapid and sensitive method for the quantitation of microgram quantities of protein utilizing the principle of protein-dye binding. *Anal. Biochem.*, **72**, 248–254.

BRUHN, S.L., PIL, P.M., ESSIGMAN, J.M., HOUSMAN, D.E. & LIPPARD, S.J. (1992). Isolation and characterisation of human cDNA clones encoding a high mobility group box protein that recognizes structural distortions to DNA caused by binding of the anticancer agent cisplatin. *Proc. Natl Acad. Sci. USA*, **89**, 2307–2311.

CHAO, C.C.-K., LEE, Y.-L., CHENG, P.-W. & LIN-CHAO, S. (1991*a*). Enhanced host cell reactivation of damaged plasmid DNA in HeLa cells resistant to cis-diamminedichloroplatinum(II). *Cancer Res.*, **51**, 601–605.

CHAO, C.C., HUANG, S., HUANG, H. & LIN-CHAO, S. (1991*b*). Cross-resistance to uv radiation of a cisplatin-resistant human cell line: overexpression of cellular factors that recognise uv-modified DNA. *Molecular & Cellular Biol.*, **11**, 2075–2080.

CHAO, C.C.-K., HUANG, S.L., LEE, L.-Y. & LIN-CHAO, S. (1991*c*). Identification of inducible damage-recognition proteins that are overexpressed in HeLa cells resistant to *cis*-diamminedichloroplatinum(II). *Biochem. J.*, **277**, 875–878.

CHU, G. & CHANG, E. (1988). Xeroderma pigmentosum group E cells lack a nuclear factor that binds to damage DNA. *Science*, **242**, 564–567.

CHU, G. & CHANG, E. (1990). Cisplatin-resistant cells express increased levels of a factor that recognises damaged DNA. *Pro. Natl Acad. Sci. USA*, **87**, 3324–3327.

CLUGSTON, C.K., MCLAUGHLIN, K., KENNY, M.K. & BROWN, R. (1992). Binding of human single-stranded DNA binding protein to DNA damaged by the anticancer drug cis-diamminedichloroplatinum (II). *Cancer Res.* (in press).

COVERLEY, D., KENNY, M.K., MUNN, M., RUPP, W.D., LANE, D. & WOOD, R.D. (1991). Requirement for the replication protein SSB in human DNA excision repair. *Nature*, **349**, 538–541.

DONAHUE, B.A., AUGOT, M., BELLON, S.F., TREIBER, D.K., TONEY, J.H., LIPPARD, S.J. & ESSIGMAN, J.M. (1990). Characterisation of a DNA damage-recognition protein from mammalian cells that binds specifically to intrastrand d(GpG) and d(ApG) DNA adducts of the anticancer drug cisplatin. *Biochemistry*, **29**, 5872–2880.

EASTMAN, A. & SCHULTE, N. (1988). Enhanced DNA repair as a mechanism of resistance to cis-diamminedichloroplatinum(II). *Biochemistry*, **27**, 4730–4734.

FICHTINGER-SCHEPMAN, A.M.J., VAN DER VEER, J.L., DEN HARTOG, J.H.J., LOHMAN, P.H.M. & REEDIJK, J. (1985). Adducts of the antitumour drug cis-diamminedichloroplatinum(II) with DNA: formation, identification, and quantitation. *Biochemistry*, **24**, 707–713.

FICHTINGER-SCHEPMAN, A.M.J., VAN DER VELDE-VISSER, S.D., VAN DIJK-KNIJNENBURG, H.C.M., VAN OOSTEROM, A.T., BAAN, R.A. & BERENDS, F. (1990). Kinetics of the formation and removal of cisplatin-DNA adducts in blood cells and tumour tissue of cancer patients receiving chemotherapy: comparison with in vitro adduct formation. *Cancer Res.*, **50**, 7887–7894.

GARNER, M.M. & REVZIN, A. (1981). Gel retardation analysis of nucleic acid-protein interactions. *Nucleic Acids Res.*, **9**, 3047–3060.

HANSSON, J. & WOOD, R.D. (1989). Repair synthesis by human cell extracts in DNA damaged by cis- and trȧns-diamminedichloroplatinum(II). *Nucleic Acids Res.*, **17**, 8073–8091.

HILLS, C.A., KELLAND, L.R., ABEL, G., SIRACKY, J., WILSON, A.P. & HARRAP, K. (1989). Biological properties of ten human ovarian carcinoma cell lines: calibration *in vitro* against four platinum complexes. *Br. J. Cancer*, **59**, 527–534.

LAI, G., OZOLS, R.F., SMYTH, J.F., YOUNG, R.C. & HAMILTON, T.C. (1988). Enhanced DNA repair and resistance to cisplatin in human ovarian cancer. *Biochem. Pharmacol.*, **24**, 4597–4600.

LAEMMLI, U.K. (1970). Cleavage of structural proteins during the assembly of the head of bacterophage T4. *Nature*, **227**, 680–685.

LILLEY, D.M.J. (1992). HMG has DNA wrapped up. *Nature*, **357**, 282–283.

MCLAUGHLIN, K., COREN, G., MASTERS, J. & BROWN, R. (1992). Binding activities of *cis*-platin-damage recognition proteins in human tumour cell lines. *Int. J. Cancer* in press.

MISTRY, P., KELLAND, L.R., ABEL, G., SIDHAR, S. & HARRAP, K.R. (1991). The relationships between glutathione, glutathione-S-transferase, and cytotoxicity of platinum drugs and melphalan in eight human ovarian carcinoma cell lines. *Br. J. Cancer*, **64**, 215–220.

PATTERSON, M. & CHU, G. (1989). Evidence that Xeroderma Pigmentosum cells from complementation group E are deficient in a homologue of yeast photolyase. *Mol. Cell Biol.*, **9**, 5105–5112.

PIL, P.M. & LIPPARD, S.J. (1992). Specific binding of chromosomal protein HMG1 to DNA damaged by the anticancer drug cisplatin. *Science*, **256**, 234–237.

REED, E., OZOLS, R.F., TARONE, R., YUSPA, S.H. & POIRIER, M.C. (1987). Platinum-DNA adducts in leukocyte DNA correlate with disease response in ovarian cancer patients receiving platinum-based chemotherapy. *Proc. Natl Acad. Sci. USA*, **84**, 5024–5028.

RICE, J.A., CROTHERS, D.M., PINTO, A.L. & LIPPARD, S.J. (1988). the major adduct of the antitumour drug cis-diamminedichloroplatinum(II) with DNA bends the duplex by $\approx$40° toward the major groove. *Proc. Natl Acad. Sci. USA*, **85**, 4158–4161.

ROBERTS, J.J., KNOX, R.J., PERA, M.F., FRIEDLOS, F. & LYDALL, D.A. (1988). The role of platinum-DNA interactions in the cellular toxicity and anti-timour effects of platinum coordination compounds. In: *Platinum and Other Metalk Coordination Compounds in Cancer Chemotherapy*, Nicolini, M. (ed.), Martinus Nijhoff Publishing, Boston.

ROBINS, P., JONES, C.J., BIGGERSTAFF, M., LINDAHL, T. & WOOD, R.D. (1991). Complementation of DNA repair in xeroderma pigmentosum group A cell extract by a protein with affinity for damaged DNA. *EMBO J.*, **10**, 3913–3921.

TEYSSIER, J., BERNARD, J., FERRE, D., DA SILVA, J. & BETTAN-RENAOUD, L. (1989). Drug related chromosomal changes in chemoresistant human ovarian carcinoma cells. *Cancer Genet. Cytogenet.*, **39**, 35–43.

TONEY, J.H., DONAHUE, B.A, KELLETT, P.J., BRUHN, S.L., ESSIGMAN, J.M. & LIPPARD, S.J. (1989). Isolation of cDNAs encoding a human protein that binds selectively to DNA modified by the anticancer drug cis-diamminedichloroplatinum(II). *Proc. Natl Acad. Sci. USA*, **86**, 8328–8332.

TOTHILL, P., MATHESON, L.M., SMYTH, J.F. & MCKAY, K. (1990). Inductively coupled plasma mass spectrometry for the determination of platinum in animal tissues and a comparison with atomic absorption spectrometry. *J. Analytical Atomic Spectrometry*, **5**, 619–622.

VAN HOUTEN, B. (1990). Nucleotide excision repair in Escherichia coli. *Microbiological Reviews*, **54**, 18–51.